# Intraoperative angioembolization in the management of pelvic-fracture related hemodynamic instability

**DOI:** 10.1186/1752-2897-5-6

**Published:** 2011-05-13

**Authors:** Robert A Cherry, David C Goodspeed, Frank C Lynch, John Delgado, Spence J Reid

**Affiliations:** 1 University of Wisconsin Hospital & Clinics Department of Orthopaedics & Rehabilitation 1685 Highland Avenue Madison, WI 53705 (608) 263-9456, USA; 2 Penn State Hershey Medical Center, Department of Radiology 500 University Drive Hershey, PA 17033, USA; 3 University Orthopaedic Associates, LLC 4810 Belmar Boulevard, Suite 102 Wall, NJ 07753, USA; 4 Penn State Hershey Medical Center, Department of Orthopedics and Rehabilitation 500 University Drive Hershey, PA 17033, USA

## Abstract

**Background:**

This case series report discusses patients presenting with hemorrhage and hemodymanic compromise due to severe pelvic fractures and undergoing intraoperative angioembolization (IAE) with other resuscitative procedures.

**Methods:**

We used portable digital subtraction fluoroscopy units for IAE in patients with severe pelvic hemorrhage and hemodynamic instability (5/03-4/09). Data was collected on demographics, injury severity, resource utilization, and outcomes at our Level 1 trauma center.

**Results:**

There were 6,538 adult admissions with 912 having pelvic fractures and 65 of these undergoing pelvic angioembolization. Twelve hemodynamically compromised patients (10 males, 2 females) had intraoperative pelvic angiography (age: 22-79 years; mean 51.3 ± 17.4). Injury severity score (ISS) was 37.5 ± 8.4 (22-50). Mean emergency department (ED) length of stay (LOS) was 57.4 min ± 47.9 with 10 patients transported directly to the OR and 2 to the SICU prior to OR. Ten of 12 patients underwent exploratory laparotomy followed by angioembolization. Mortality was 50%. Among the 6 survivors (ISS 22 - 50), all had a pre-op CT scan, five had an initial base deficit <13, and four were transfused ≤ 6 units pre-incision/pre-procedure. Four of the 6 survivors had unilateral embolization. In contrast, all 6 non-survivors (ISS 29-41) required massive transfusion prior to OR (>6 units PRBCs) with 4 having a based deficit >13. Three of these patients bypassed CT and five underwent bilateral internal iliac embolization (BIIE).

**Conclusions:**

IAE for severe pelvic hemorrhage can be successfully performed concurrently with exploratory laparotomy, pelvic packing or other resuscitative procedures. Patients most likely to benefit have a base deficit <13, and do not require massive transfusion prior to IAE or suffer from a vertically unstable pelvis fracture.

## Introduction

Angioembolization has long been used to control pelvic hemorrhage in hemodynamically compensated trauma patients. Mobile angiography has now been reported in the Emergency Department (ED) to facilitate immediate control of hemorrhage resulting from severe pelvic fractures [1,2]. The use of this approach in the operating room has not been widely described in the literature. In this case series report, we described our experience with hemodynamically unstable patients due to hemorrhage from severe pelvis fractures and who underwent selective and non-selective angiography and embolization in the operating room with other resuscitative procedures. The purpose of this study was to explore the feasibility and delineate the appropriate indications for intraoperative pelvic embolization based on a comparison of the clinical presentation between survivors versus non-survivors.

## Materials and methods

This is a descriptive case series report involving adult patients ages 18 years and older who underwent intraoperative selective and nonselective angiography from May 2003 through April 2009. We used portable digital subtraction fluoroscopy units (OEC 9800, General Electric Healthcare Inc., Tampa, Florida) for intra-operative angiography and embolization in patients with severe pelvic hemorrhage at our Level 1 trauma center. Patients were placed on an OSI surgical imaging table (Mizuhosi; Union City California) and a mobile angiography cart was made available. All embolization procedures were performed to occlusion using Gelfoam slurry by a fellowship trained interventional radiologist. Data was collected on demographics, injury severity, resource utilization, and outcomes using the trauma registry (Collector, Digital Innovations, Forrest Hill, Maryland) and the electronic medical record (Cerner Corporation, Kansas City, Missouri). Pelvic fractures were classified based on definitions used by the Orthopedic Trauma Association (OTA). Mean variables were described using standard deviation. Institutional Review Board approval was obtained. Written clinical guidelines for the use of intraoperative pelvic angioembolization have been in place since August of 2004.

## Results

From May 2003 to April 2009, there were 912 pelvic fractures in 6,508 adult trauma patients meeting criteria for the Pennsylvania Trauma Outcomes Study (PTOS). PTOS criteria include those patients admitted for at least 48 hours and were treated with a diagnosis of trauma (ICD-9-CM injury codes 800-995). A total of 65 pelvic angiograms were performed or 1% of all PTOS patients. Twelve hemodynamically unstable patients (10 males, 2 females) over the 6-year period underwent intraoperative pelvic angiography (age 22 to 79 years; mean 51.3 ± 17.4). Mean ISS was 37.5 ± 8.4 (22 - 50) with 8 patients receiving > 6 units of pre-OR (or pre-incision/pre-procedure). The mean base deficit was 12.2 ± 6.1 (3.2 - 20.9). ED LOS was 57.4 min ± 47.9 with 10 patients transported to OR and two to SICU prior to intraoperative angiography. None of the patients were considered eligible for transport to the angiography suite secondary to hemodynamic compromise. The radiographic contrast dose for all 12 patients was 113 ± 59.9 ml (25-210). There were no angiography related complications. Mortality was 50%.

Among the six survivors (ISS 22-50; ages 22-79), five underwent laparotomy with angiography performed prior to surgery in one patient (See Table 1). All had a pre-op CT scan and four had an initial base deficit < 13. Four were transfused ≤ 6 units pre-procedure/pre-incision and five with an INR < 1.7. Two patients were initially admitted to the surgical intensive care unit; the first patient was transferred from the emergency department, and the second was a direct admission from an outside hospital. Four of the five surviving patients who were direct admits to the ED had an ED length of stay > 60 minutes. Only one patient had a vertically unstable pelvic fracture (OTA 61C). Two patients had bilateral nonselective internal iliac artery embolization (BIIE) using gel foam slurry. Hospital length of stay ranged from 14-55 days with three patients discharged to a rehabilitation center and three to a skilled nursing facility (SNF).

**Table 1:** Characteristics of survivors undergoing intraoperative pelvic angiogram.

	Survivors					
	
	Patient #					
	
	1	2	3	4	5	6
Mechanism	MVC	MVC	MVC	Other blunt	MVC	MVC
Age	68	55	22	53	79	49
Gender	Male	Male	Male	Female	Male	Female
ISS	29	50	41	22	38	50
Fracture Type	Left both column acetabular fracture	Left iliac wing fracture	Right both column acetabular fracture	Sacral compression fracture	Left ilium and sacroiliac joint	Sacral compression fracture
OTA	L 62C3	L 61C1.1	R 62C3	L61B-2.1	L61A-2.2	L61B-2.1
ED LOS (min)	> 60	> 60	>60	25	ED-to-ED Transfer	> 60
Hospital LOS	14	34	26	28	55	18
ICU LOS	11	34	21	6	22	11
Vent Days	10	34	20	5	16	7
Pre-OR PRBC	4 units	4 units	6 units	4 units	> 6 units	> 6 units
Initial B.D.	3.2	7.2	5.6	> 13.0	6.1	12.2
Initial INR	1.04	1.66	> 1.7	1.46	1.12	1.02
Pre-OR CT	Yes	Yes	Yes	Yes	Yes	Yes
Exp Lap	Yes	Yes	No	Yes	Yes	Yes
Angio First	No	No	Yes	No	No	No
Disposition	Rehab	SNF	Rehab	SNF	SNF	Rehab

Among the six non-survivors (ISS 29-41; ages 29-72), 5 of the 6 patients underwent laparotomy first with intraoperative angiography performed prior to laparotomy in one patient (See Table 2). All required massive transfusion (> 6 units PRBC) prior to incision/procedure in the OR with an initial INR >1.7 in two patients. Four patients had an initial base deficit > 13. Two out of the six patients had an ED length of stay > 60 minutes. Five out of the six patients had a vertically unstable pelvis fracture (OTA 61C). Five out of six underwent bilateral internal iliac embolization (BIIE) using Gelfoam slurry. Three patients bypassed CT and had an ED length of stay from 17-20 minutes. All three of these patients had an exploratory laparotomy prior to angiography. Two of the three patients with a preoperative CT scan had a severe base deficit on presentation (16.9 and 20.9). The third patient with a preoperative CT scan suffered multiple bilateral cerebellar contusions and intraventricular blood, and was eventually pronounced brain dead. Five out of six patients expired within 24 hours. Two patients expired in the OR from internal iliac artery injuries despite a technically successful BIIE.

**Table 2:** Characteristics of non-survivors undergoing intraoperative pelvic angiogram.

	Non-Survivors					
	
	Patient #					
	
	7	8	9	10	11	12
Mechanism	MCC	MVC	MVC	Pedestrian	MCC	MCC
Age	57	42	29	56	33	72
Gender	Male	Male	Male	Male	Male	Male
ISS	34	41	41	41	34	29
Fracture Type	Sacral fractures	Sacral compression fracture	Left iliac wing fracture	Right sacral fracture, left acetabular fracture	Sacral fracture	Sacroiliac joint fracture
OTA	61C2.2	R 61B3.1	L 61C1.1	R 61C1.3, L 62B2.3	R61C1.3	L61C1.2
ED LOS (min)	26	> 60	> 60	20	15	17
Hospital LOS	1	1	1	12	1	1
ICU LOS	0	1	1	12	0	1
Vent Days	1	1	1	12	1	1
Pre-OR PRBC	> 6 units	> 6 units	> 6 units	> 6 units	> 6 units	> 6 units
Initial B.D.	> 13.0	> 13.0	8.5	> 13.0	12.3	> 13.0
Initial INR	1.43	> 1.7	1.33	> 1.7	1.07	1.04
Pre-OR CT	Yes	Yes	Yes	No	No	No
Exp Lap	Yes	Yes	No	Yes	Yes	Yes
Angio First	No	Yes	No	No	No	No
Disposition	N/A	N/A	N/A	N/A	N/A	N/A

**Figure 1:**
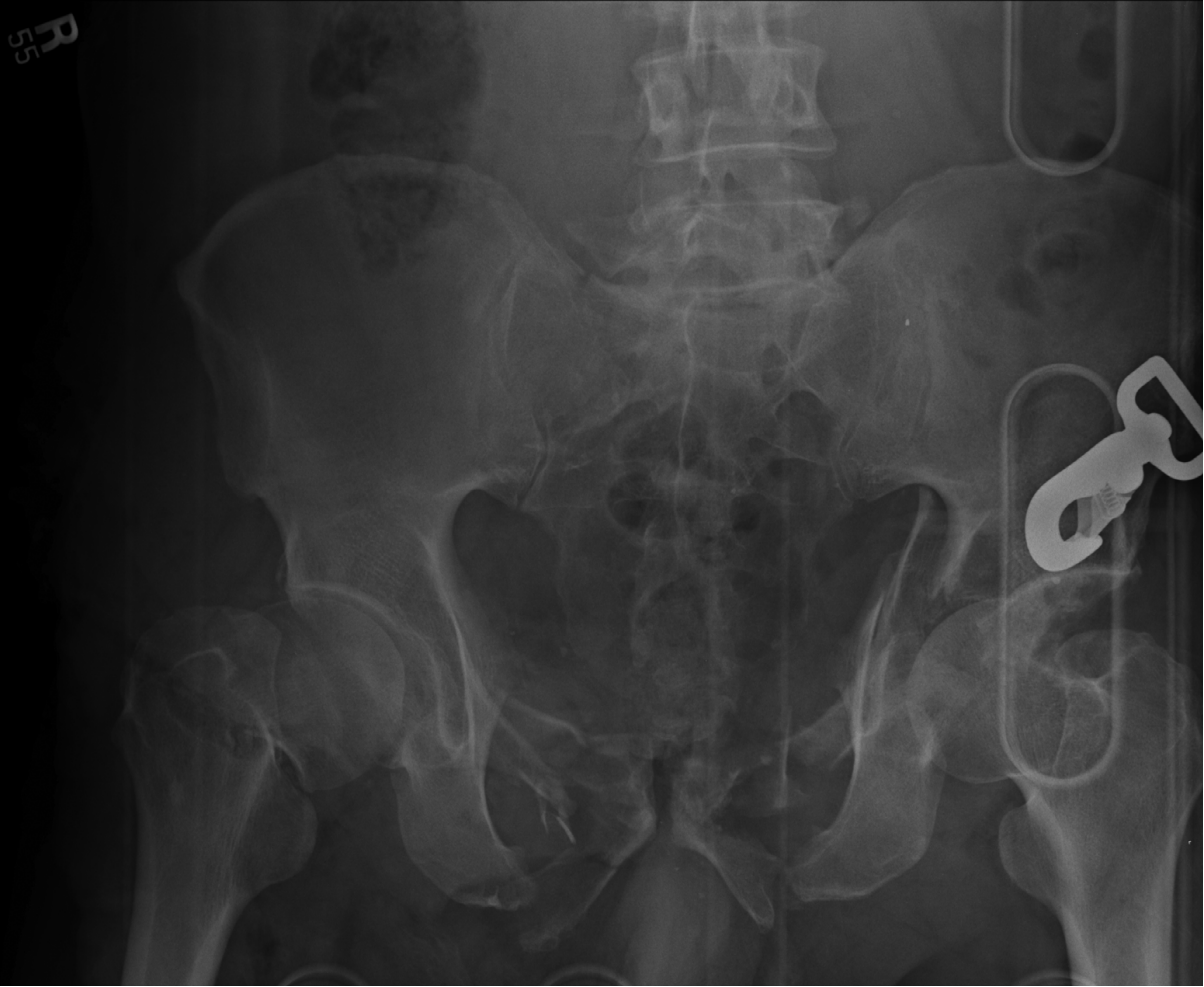
**Patient # 10 - Pelvis X-Ray**.

**Figure 2:**
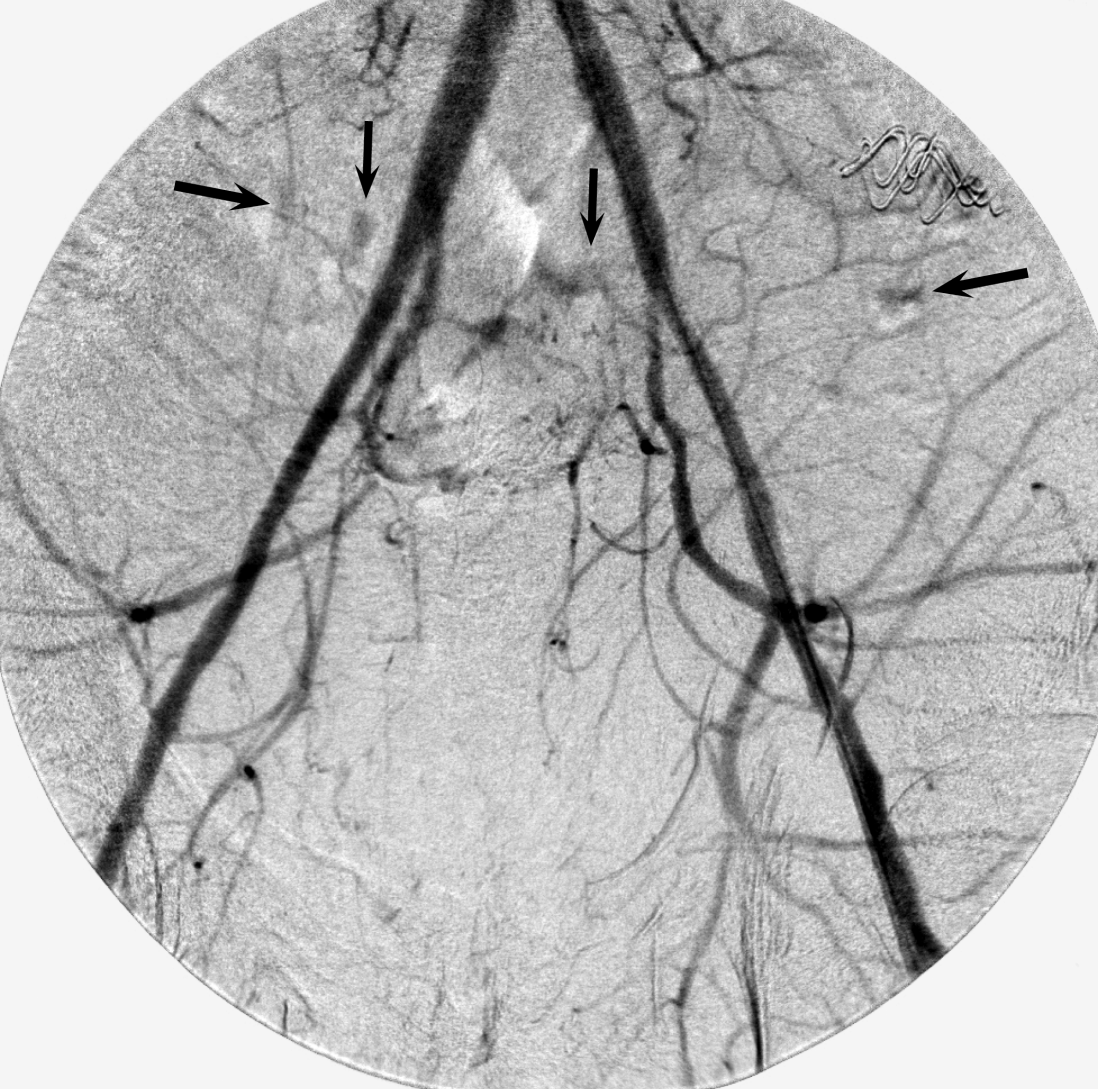
**Patient # 10 - Intraoperative Pelvic Angiogram**. Arrows point to areas of active contrast extravasation.

Figure 1 is the pelvis x-ray for Patient # 10. This plain film demonstrates a right displaced femoral neck fracture and a left acetabular fracture. Comminuted superior and inferior pubic rami fractures are noted bilaterally. A fracture through the right sacrum is also seen, along with a fracture of the left L5 transverse process. Figure 2 shows active extravasation of contrast from numerous branches of both the anterior division and posterior divisions of both the right and left internal iliac arteries, suggesting multiple bleeding sites. Both internal iliac arteries were embolized with Gelfoam to occlusion.

Table 3 demonstrates the findings at angiography, including the vessels embolized and whether BIIE was necessary. In three of the cases, the bleeding vessel was not identified. One patient required surgical ligation of the right internal iliac artery. The other two underwent either empiric BIIE or unilateral internal iliac embolization based on the fracture pattern and continued clinical evidence of bleeding from the pelvis. Limitations in the resolution of intraoperative fluoroscopy compared to the angiography suite may account for the lack of findings.

**Table 3:** Intraoperative pelvic angioembolization - findings and procedure.

Patient #	Bleeding Vessel	Vessel Embolized	Bilateral Embolization
1	Not identified (distal small vessel irregularity)	Left Internal iliac	No
2	Left Internal iliac anterior/posterior division; right lateral sacral	left internal iliac/R lateral sacral	Yes
3	Right internal iliac anterior/posterior division	Right Internal iliac	No
4	Bilateral anterior division branches internal Iliac	Left internal iliac anterior/right vesicle artery/right obturator (bilateral anterior division branches)	Yes
5	Left internal Iliac, anterior and posteriors divisions	Left internal iliac	No
6	Not identified	Left internal iliac	No
7	Left Internal illiac anterior division, right internal Iliac	Bilateral internal iliac	Yes
8	Not identified	Bilateral internal iliac	Yes
9	right branch anterior division (vesical); left superior gluteal, iliolumbar	Right internal iliac anterior division/Left internal iliac posterior division	Yes
10	Multiple branches bilateral internal Iliac	Bilateral internal iliac	Yes
11	Not identified	None (surgical ligation right internal illiac)	N/A
12	Right internal iliac	Bilateral internal iliac	Yes

## Discussion

Intra-operative angiography and embolization in patients with severe pelvic hemorrhage can be successfully and safely performed concurrently with exploratory laparotomy, pelvic packing or other resuscitative procedures. However, the procedure is best selected for patients in whom intraoperative angiography is likely to impact survival.

Patients with a base deficit of less than 13, as well as those not requiring massive transfusion or suffering from a vertically unstable pelvis fracture (OTA 61C), are most likely to benefit from the procedure. This is also consistent with work by Wong and colleagues in which the rate of blood transfusion prior to embolization was predictive of mortality [3]. In their study, death was associated with 11.3 ± 11.0 units of blood transfused per hour versus 3.2 ± 1.9 units per hour in survivors. Fangio and associates also found that the rate of blood transfusion was significantly higher in nonsurvivors [4].

Manual compression with pelvic binders should be performed early during the resuscitation in appropriate patients. Based on our experience, total ED length of stay for these patients should also be kept under one hour whenever possible. The decision to perform CT scan prior to or after embolization is largely dependent on the ability to achieve reasonable hemodynamic compensation. In a clinical algorithm for managing hemodynamically unstable patients with pelvic fractures, Jeske and coworkers were able to obtain pre-embolization CT images in 24 out of 42 patients [5].

When possible, embolization should be performed prior to laparotomy in hemodynamically stable patients since this has been shown to reduce mortality [6]. However, all of the patients in our study were hemodynamically compromised, and five out of six patients in each group (survivors and non-survivors) required laparotomy prior to angioembolization in. This is one of the major advantages in bringing pelvic embolization to the operating room in this subset of patients. Furthermore, the success rate in pelvic angioembolization in stopping arterial hemorrhage has been reported to be 85-100% [4].

Other interventions, such as preperitoneal pelvic packing, should be considered in patients who are more physiologically distressed. Repeat angiography may also be needed for persistent hypotension and acidosis [7]. Hemodynamically stable patients without the need for immediate concurrent operative procedures should continue to have intraoperative embolization in the interventional radiology suite where resolution of the images is considered superior and may allow a more selective procedure.

There are uncommon but serious complications that may result from either unilateral of bilateral internal iliac artery angioembolization [8]. These include thigh or buttock claudication [9-14], gluteal necrosis [15-18], rectal necrosis [17], gluteal compartment syndrome [19], sexual dysfunction [11-14,20], and lower extremity paresis [17,21]. One of our patients suffered unilateral lower extremity ischemia in the context of severe injuries to that limb. While this may have been due to the underlying injury, it is possible that embolization material, intended for the internal iliac artery was inadvertently allowed to reflux into the external iliac artery and distally. We suspect that there might be a higher incidence of non-target embolization when this procedure is performed in the operating room using portable imaging equipment that has lower special and contrast resolution as well as a smaller field of view. Hybrid operating rooms with fixed high quality angiographic equipment are becoming more common at many institutions. The increased imaging quality made available in these rooms in theory would increase the likelihood of successful embolization while reducing the incidence of non-target embolization.

This is the largest case series report involving intraoperative pelvic angiography. Nevertheless, there are several limitations involving the study. First, caution should be exercised in interpreting the data on a relatively small patient population involving a single center, retrospective study. Second, high-quality resolution of intraoperative angiographic images is not as superior as those performed in the interventional radiology suite. Although there were no angiography related complications, we do not know how the limited resolution of those images may or may not have impacted these critically injured patients. Finally, this report does not directly compare intraoperative pelvic embolization with other modalities that might be used in the hemodynamically unstable patient, such as preperitoneal packing.

## Conclusions

We believe that the operating room is the optimal environment for a multi-modality approach in treating severe and complex pelvic fractures associated with hemodynamic instability and multisystem injuries in selected patients. Intra-operative angioembolization in patients with severe pelvic hemorrhage can be successfully performed concurrently with exploratory laparotomy, pelvic packing or other resuscitative procedures. Clinical outcomes may be improved when combined surgical and percutaneous approaches are executed in operating rooms with high quality fixed imaging equipment. Those patients most likely to benefit have a base deficit of less than 13; are not requiring massive transfusion prior to the start of their intraoperative procedure; and do not have a vertically unstable pelvis fracture.

## List of Abbreviations

IAE: intraoperative angioembolization; BIIE: bilateral internal iliac embolization; LOS: length of stay; ED: Emergency Department; PTOS: Pennsylvania Trauma Outcomes Study; SNF: skilled nursing facility; ISS: injury severity score

## Competing interests

The authors declare they have no competing interests.

## Authors' contributions

RC (1) substantial contributions to conception and design, and analysis and interpretation of data; and (2) drafting the article or revising it critically for important intellectual content; and (3) final approval of the version to be published. DG (1) substantial contributions to acquisition of data; and (2) revising the article critically for important intellectual content; and (3) final approval of the version to be published. FL (1) substantial contributions to analysis and interpretation of data; and (2) revising the article critically for important intellectual content; and (3) final approval of the version to be published. JD (1) substantial contributions to acquisition of data; and (2) revising the article critically for important intellectual content; and (3) final approval of the version to be published.JSR (1) substantial contributions to conception and design, acquisition of data, and interpretation of data; and (2) drafting the article or revising it critically for important intellectual content; and (3) final approval of the version to be published.
